# Neuromuscular effects suggest that imagery engages motor components directly – a commentary on Frank et al. (2023)

**DOI:** 10.1007/s00426-024-01943-y

**Published:** 2024-03-14

**Authors:** Waltraud Stadler, Joachim Hermsdörfer

**Affiliations:** Department Health and Sport Sciences, School of Medicine and Health, Technical University of Munich, Munich, Germany

## Abstract

Not applicable.

The perceptual-cognitive scaffolding account proposes that learning through imagery acts solely on the perceptual-cognitive level, implying a clear separation of cognitive from motor processes. An indirect impact on motor processes is considered possible if the scaffold is already linked to motor components, i.e., for actions with a certain level of familiarity. The theory allows clear predictions for empirical studies which permits substantiation on a broad body of evidence. However, it does not seem to apply to neuromuscular effects of imagery. These are, for instance, shown in studies combining transcranial magnetic stimulation (TMS) with electromyography (EMG) to measure motor evoked potentials during imagery (Grosprêtre et al., 2016 for a review) suggesting that the relevant motor pathways are immediately activated during imagination of a motor act. Activation is specific to relevant muscles (Mihelj et al., 2021) and can be modulated by the imagined force level (Mizuguchi et al., 2013). Moreover, motor imagery training can increase muscle force (Reiser et al., 2011; Smith et al., 2003; Yue & Cole, 1992). In their seminal work, Yue and Cole (1992) reported a 22% increase of maximal voluntary contraction force in the left abductor digiti minimi after imagery training which is not much less than 30% increase measured after overt practice (Yue & Cole, 1992). Frank and colleagues briefly refer to this literature, suggesting that also here, imagery changes “cognitive elements” which lead to neuromuscular facilitation (Frank et al., 2023, p. 7). This brief explanation seems unsatisfying, given that the activation of motor pathways does not just seem to be “motor outflow” (Bach et al., 2022, p. 5) but is systematic enough to induce plasticity. There is evidence that imagery can induce plastic changes in the primary motor cortex (M1) (Yoxon & Welsh, 2019) and on the level of spinal interneurons (Grosprêtre et al., 2019; Wieland et al., 2022). Such modulations are indeed supportive of the specification of motor commands through imagery (Heuer, 1989), which Frank and colleagues are arguing against. Notably, however, the perceptual-cognitive scaffolding account mostly refers to learning of a new motor skill whereas imagery interventions for strength increase mostly involved very different task requirements. In imagined strength training regimes the same defined muscles were addressed repeatedly in simple mono-articular movements or isometric contractions (Reiser et al., 2011; Smith et al., 2003; Yue & Cole, 1992). For instance, in the study by Yue and Cole abduction force of the left little finger was trained by repeatedly imagining only the finger abduction movement in a force transducer while the rest of the hand and the other fingers were restricted (Yue & Cole, 1992). Reiser an colleagues used maximal isometric contractions in strength exercises (Reiser et al., 2011). Such strength training differs from skill learning with respect to the kind of cognitive processes it implies. To achieve force gains through imagery, cognitive guidance is undoubtedly required (Glover & Baran, 2017) but might play a rather general role of attention focus (Martel & Glover, 2023). In contrast, in skill learning tasks referenced by Frank and colleagues, such as the serial reaction time task, improvement is mainly achieved through cognitive strategies and consequently these are primarily addressed in imagery. Consequently, here, imagery likely involves abstract action representations and improvement results from learning of, e.g., structural elements such as memorizing a number / key sequence. Such tasks do not address specific muscle contractions and modulations at the spinal level might not be required. Frank and colleagues argue that tasks with a strong cognitive component benefit more from imagery. We agree that not every action-imagery learning task might induce plasticity on the neuromuscular level. However, optimizing paradigms to address motor programs in imagery is highly relevant as improvement or conservation of muscle force through imagery bears a huge potential for motor neurorehabilitation (Bassolino et al., 2014).

An aspect to consider is the limited awareness of motor processes (Frith et al., 2000). The link from perceptual events to appropriate motor commands is implicit and we can only access motor processes through sensory feedback. In imagery, in contrast to overt action, external feedback is not constantly available and has to be substituted by memorized effects (Bach et al., 2022), which underlines the importance of perceptual aspects and executive guidance (Martel & Glover, 2023). Moreover, depending on modality and task, perception can involve motor components (Frith et al., 2000) as action planning can activate the visual cortex (Bach et al., 2022). The notion of a strict separation between cognitive imagery and neuromuscular control could be replaced by a variable, i.e., task-dependent, overlap of cognitive with motor processes. Various ways in which perceptual and motor functions overlap are outlined by Bach and colleagues in this issue and we may add the evolutionary history of the human brain (Cisek, 2019). From such a perspective, cognitive functions are implemented in formerly sensorimotor brain circuitries and employ functions which originally developed in a pragmatic context. Regarding the fronto-parietal network, which is reliably activated during motor imagery (Kraeutner et al., 2022; Lebon et al., 2018) and action prediction (Balser et al., 2014; Stadler et al., 2011), several authors (Ptak et al., 2017; Schubotz, 2007) have suggested that it has the function of manipulating, maintaining and transforming events in mind. During ontogenetic development, its functions evolve from elementary motor planning to a cognitively flexible system (Ptak et al., 2017). We assume that transitions between perceptual-cognitive and motor processes are gradual and that functional contributions are determined by the task.

Modalities of imagery depend on task requirements, too, and the composition of the neural network underlying action observation and imagery is highly sensitive to task instructions (Lebon et al., 2012; Stadler et al., 2011; Zentgraf et al., 2005). We interpret it as indicative for direct neuromuscular activation that kinesthetic imagery involves the primary motor cortex (M1) typically more than visual imagery (Lebon et al., 2012, 2018) and that plasticity in the motor system and force gains benefit best from this same kinesthetic imagery modality (Grosprêtre et al., 2016). Assuming task specific modulations we must necessarily expect certain differences between imagery and overt action.

To conclude, the notion of a task-specific neural overlap between motor and cognitive components in imagery is compatible with theories on neural processes of imagery. First, in line with the simulation account (Jeannerod, 2001), we assume that actions are implemented in the same circuits when imagined, where functional equivalence might exist on a local level. Highlighting the role of task instructions, we assume that actions which strongly involve cognitive components will do so during imagery and overt execution alike. Motor programs can benefit directly from imagery through the activation of such common neural mechanisms. However, activity during imagery is not identical to overt action which might not least be due to inhibitory circuits which prevent imagined movement from being executed, as described by inhibition theories of motor imagery (Rieger et al., 2017). We expect that imagery most effectively acts on the neuromuscular level when a single muscle is addressed repeatedly through kinesthetic imagery as this modality has been shown to increase the likelihood of primary motor cortex activation. This assumption requires further clarification as well as the question under which conditions skill learning involves motor components.

## Data Availability

Not applicable. This commentary does not use original data or any materials.

## Declarations

### Clinical trial number

Not applicable. The manuscript does not contain clinical studies or patient data.
